# Evaluation of an integrated system for classification, assessment and comparison of services for long-term care in Europe: the eDESDE-LTC study

**DOI:** 10.1186/1472-6963-13-218

**Published:** 2013-06-15

**Authors:** Luis Salvador-Carulla, Javier Alvarez-Galvez, Cristina Romero, Mencia R Gutiérrez-Colosía, Germain Weber, David McDaid, Hristo Dimitrov, Lilijana Sprah, Birgitte Kalseth, Giuseppe Tibaldi, Jose A Salinas-Perez, Carolina Lagares-Franco, Maria Teresa Romá-Ferri, Sonia Johnson

**Affiliations:** 1 Centre for Disability Research and Policy Faculty of Health Sciences, University of Sydney, 75 East St Lidcombe, Sydney, NSW 2141, Australia; 2 Scientific Association PSICOST, Cadiz, Jerez de la Frontera, Spain; 3 Área de Salud y Política Social, Universidad Loyola Andalucía, Seville, Spain; 4 Center for Teaching and Learning, University of Vienna, Vienna, Austria; 5 LSE Health and Social Care, London School of Economics and Political Sciences, London, UK; 6 Public Health Association, Sofia, Bulgaria; 7 Sociomedical Institute, Scientific Research Centre of the Slovenian Academy of Sciences and Arts, Ljubljana, Slovenia; 8 Department of Health Research, SINTEF Technology and Society, Trondheim, Norway; 9 Centro Studi e Ricerche in Psichiatria ASLT02, Torino, Italy; 10 Mental Health Sciences Unit, University College London, London, UK; 11 Área de Estadística e Investigación Operativa, Universidad de Cádiz, Cádiz, Spain; 12 Departamento de Enfermería, Universidad de Alicante, Alicante, Spain

**Keywords:** Health service research, Health system research, Healthcare terminology, Healthcare taxonom, Healthcare instrument

## Abstract

**Background:**

The harmonization of European health systems brings with it a need for tools to allow the standardized collection of information about medical care. A common coding system and standards for the description of services are needed to allow local data to be incorporated into evidence-informed policy, and to permit equity and mobility to be assessed. The aim of this project has been to design such a classification and a related tool for the coding of services for Long Term Care (DESDE-LTC), based on the European Service Mapping Schedule (ESMS).

**Methods:**

The development of DESDE-LTC followed an iterative process using nominal groups in 6 European countries. 54 researchers and stakeholders in health and social services contributed to this process. In order to classify services, we use the minimal organization unit or “Basic Stable Input of Care” (BSIC), coded by its principal function or “Main Type of Care” (MTC). The evaluation of the tool included an analysis of feasibility, consistency, ontology, inter-rater reliability, Boolean Factor Analysis, and a preliminary impact analysis (screening, scoping and appraisal).

**Results:**

DESDE-LTC includes an alpha-numerical coding system, a glossary and an assessment instrument for mapping and counting LTC. It shows high feasibility, consistency, inter-rater reliability and face, content and construct validity. DESDE-LTC is ontologically consistent. It is regarded by experts as useful and relevant for evidence-informed decision making.

**Conclusion:**

DESDE-LTC contributes to establishing a common terminology, taxonomy and coding of LTC services in a European context, and a standard procedure for data collection and international comparison.

## Background

Long-Term Care (LTC) is a blanket term that “brings together a range of services for persons who are dependent on help with basic activities of daily living over an extended period of time” [1]. This range comprises ‘medical and/or social services designed to help people who have disabilities or chronic care needs. Services may be short or long-term and may be provided in a person’s home, in the community, or in residential facilities’ (US Dept of Health). However it should be noted that, at present, Member States of the European Union use a variety of definitions of LTC that do not always concur [2].

Comparing health services across different countries is very difficult, especially for services that aim to deliver Long-Term Care. Thus far, international service comparison has largely failed to provide satisfactory information for health planning in areas as diverse as mental health [3], ageing [4], or services for persons with disabilities in Europe [5]. These comparison problems can be attributed to factors that include: (1) the influence of local history in the development of specific service models; (2) differences in organizational structure; (3) increasing complexity of service networks [6]; (4) terminological inconsistencies (services with the same name perform different activities and *vice versa*); (5) problems in the definition of the target population for whom the services are designed; (6) lack of an international classification system to facilitate standard coding of services across different settings.

Terminological variability is found across all types of health service, from day hospitals to rehabilitation centers, and we even lack an operational definition of ‘service’ that can be applied to comparative health service research [7]. ‘Service’ is used in several senses in the System of Health Accounts (SHA 2.0) [8]. In this context the term can be used to describe provider organizations, a combined arrangement of functions, programmes and goods (e.g. Common Listing of Services of the Spanish National Health Service) [9], a physical facility, or an organizational unit. Even in the latter restricted use, this term can be used to describe the organization of inputs at micro-level (i.e. at an individual local facility), at meso-level (e.g. a general hospital in a small catchment area) or at macro-level (e.g. a health maintenance organization working at regional or national level or a public health preventive programme). Further, the lack of an international taxonomy in health service research impedes comparisons of like with like. Thus lists of services based on official names group highly structured ‘services’ together with simple ‘clinical units’ or even with care activities, as if they were comparable units of analysis. The lack both of international units of analysis and of a health service taxonomy generates an incommensurability bias in the whole area of health services research [10]. It is also a major impediment to international comparability and to the practical use of health service data for evidence-informed policy.

European health agencies need comparable descriptions of care to measure health equity and allow patient mobility within the European Union. The Charter of Fundamental Rights of the European Union (2000) established that ‘having access to high-quality healthcare when and where it is needed’ is a fundamental right of every European citizen. However, currently parity of access and mobility between appropriate services are difficult to assess because we do not have tools for ascertaining which services might be seen as equivalent [11,12]. Hence the development of a common coding and a standard assessment system is also relevant for services harmonization and the equitable allocation of care (resources, programmes and treatments) between the different groups and individuals in Europe, as well as for facilitating the linkage of European health [13].

The World Health Organization has also made a strong case for international service comparisons in the assessment of health care reforms [14]. The WHO Advisory Committee on Health Research has highlighted the importance for the development of usable and grounded policy recommendations of using local evidence on service availability obtained from the specific setting or area regarding which plans are being made [15,16]. Meeting the need for complementary local (meso-level) and global/national/regional information (macro-level) is one of the elements in the SUPPORT program for improving decision making about health policies and programs [17].

As a starting point, the European Psychiatric Care Assessment Team (EPCAT) initiated in the year 1994 the development of a common terminology and a standard procedure for assessment of mental health services. The underlying aim was to facilitate comparisons between different geographical areas so as to generate a robust evidence base for health planning and resource allocation. A further aim was to provide contextual information to support interpretation of research findings. The EPCAT group developed a battery of instruments for international comparison of mental health services. This battery included a brief set of indicators of small mental health area characteristics (European Socio-Demographic Schedule - ESDS) [18], a standard assessment of care activities within mental health services (International Classification of Mental Health Care - ICMHC) [19] and an instrument or schedule for coding and assessing mental health services (European Service Mapping Schedule - ESMS) [20]. Underpinning this instrument was a consensus on a standard method for service assessment and comparison in small health areas. In the subsequent years this system was used to make catchment area-based comparisons of mental health care in several countries, for example Italy [21], Spain [22], Poland [23] or Germany [24]. The system also proved its usability for international service research including comparisons of the mental health systems in Spain, Italy and Chile [25,26], and in Norway and Russia [27], as well as in a series of international studies mainly conducted in Europe [28]. Prior tools developed by our group included instruments for evaluation of services in mental health (ESMS) [20], the elderly (DESDAE) [27] and disability (DESDE) [3].

The current study has built on this preceding programme with the aim of developing a standard taxonomy for description, mapping and comparison of services for Long-Term Care (DESDE-LTC). The DESDE–LTC toolkit comprises a coding system, a glossary of terms, a service assessment instrument, a training package and a casebook. The DESDE-LTC toolkit and all the preliminary information on this project is available at the project’s website (http://www.edesdeproject.eu).

## Methods

The study has been carried out by a consortium of research organizations on health services in 6 European countries, and a series of collaborating experts. These include developers of the European Service Mapping Schedule (ESMS), as well as experts from international organizations (OECD), health agencies (national, regional and municipality levels), and experts in formal ontology and support decision systems for health decision-making, ensuring its face and content validity.

The development of the DESDE-LTC classification followed a series of related steps. First a review was conducted of the framework for coding and classification services for LTC in Europe, including a re-examination of previous instruments (ESMS, DESDE, DESDAE). Then a first draft of the new coding system and the instrument was prepared by the members of the core working group. This preliminary version included modifications of the initial coding based on the literature review and on the experience gathered in health and social service mapping in Spain after the completion of DESDE in 2006, as well as on the care needs of the broader target group (LTC), which included also acute services as these services are used by this population group.

The preliminary version was then reviewed through an iterative process which involved nominal groups in the 6 participating countries. A total of 41 European researchers and stakeholders in LTC health care and social services participated in the nominal groups, and 54 in the usability exercise which included international experts in the use of the ESMS and DESDE. The Nominal Group Technique is a more structured variation of the focus group for decision-making and planning [29]. It allows a group to achieve consensus and prioritize issues, as it retains the consensus-building benefits of the group dynamic while harnessing a range of individual views. Thus this technique allows the collection of an appropriate amount of evidence-informed local data to calibrate the instrument.

Three sessions were organized in every country with the following objectives: (a) to introduce the problems of service research and comparability of services across different geographical areas in Europe, to familiarize participants with the EPCAT approach to services research and with the DESDE-LTC coding system, and to prepare comments and amendments which were discussed at Session 2; (b) to acquaint participants with the DESDE-LTC coding system, including reviewing the aim, structure and use of the instrument, and the cut-off points provided in it; (c) to conduct the final review, prepare the final version of the classification and its toolkit and to confirm that suggestions of every nominal group have been taken account. Finally a conceptual adaptation of this preliminary version of the coding system and the instrument was made in 6 languages: English, Spanish, German, Norwegian, Slovenian and Bulgarian.

Once the final DESDE-LTC versions of the classification and its instrument were available, the usability of the coding system was analyzed according to four quality criteria: Feasibility, Consistency, Reliability and Validity [30]. The full description of the feasibility analysis is available elsewhere [31].

### Sample

This analysis was made using a PSICOST health and social services database that is made up of 1339 services, each identified as the minimal organizational set of inputs or Basic Stable Input of Care (BSIC) (see below). Table 1 describes the different units of analysis of services and their definitions including the units employed in this instrument (BSIC and MTC).

**Table 1:** Different units of analysis in health services comparison and related terms

**Descriptor**	**Complete name**	**Definition**
Facility	Health facility	A physical structure (building or dwelling) where care is provided (BSICs are delivered in care facilities).
Service	Health service (generic)	A ‘service’ is an umbrella term that is used to describe very different components of the organization of care typically at the micro-level functional system. It could refer to facilities, organizational arrangements within a facility, health deliverables such as programs and interventions or health products.
	Service delivery*	Combination of inputs into a service production process that delivers health interventions to individuals or to the community.
	Health product*	The result of the interaction of capital, labor and entrepreneurship in the production process which has the primary purpose of improving, maintaining or preventing the deterioration of the health status of persons mitigating the consequences of ill-health defined as the smallest unit with own administrative structure available within the local area (micro-organization). The range of services to be considered includes those facilities that have as specific aim any aspect of the management of health care and of the clinical and social difficulties related to it.
	Health services*	Products over which ownership rights cannot be established, although they typically generate changes in the conditions of the consuming units, including health benefits for the receiving individuals. They cannot be traded separately from their production, and they are the result of the activities of producers at the demand of consumers.
BSIC	Basic stable input of care	Is the minimal organizational unit composed by a set of inputs with temporal stability arranged for delivering health related care to a defined population in a care area. It is usually composed of an administrative unit with an organized set of structures and professionals. Within the production model of health related care (input-throughput-output), BSIC refers only to functions of care and not to other inputs (devices, facilities) or to procedures (interventions). The functions provided by this unit care can be described by the main type of care provided by the BSIC. The operational description of BSIC depends on its main characteristics (organization, staff, location and target population).
MTC	Main type of care	MTC is the main DESCRIPTOR of the ‘generic care function’ provided by the BSIC. The generic care function typically describes the principal activity carried out in the BSIC (e.g. the user sleeps in a setting where a physician is available 24 hours a day).
		MTCs have been selected in an iterative process by a series of expert groups based on the actual description of services in different Countries within the consecutive ESMS/DESDE projects.

(*) *SHA 2.0* System of Health Accounts 2.0.

The service sample mainly included services from Spain (n = 1319), since this country has a high diversity of health and social care systems in its 17 regions. Additionally, other specific services from 5 European countries were incorporated to increase variability of the sample (n = 20)^a^. To extend the description of types of services, the dataset for mental health care (n = 1275) was extended with 64 services for other LTC groups (intellectual disabilities, physical disabilities, elderly and other chronic conditions). This allowed inclusion of as broad as possible a range of services in the reliability and validity exercises. The training casebook included 21 different cases from all six participating countries [31].

### Feasibility analysis

An *ad-hoc* 25-item feasibility questionnaire with a 5-point Likert scale (where 1 = “best/highest” and 5 = “worst/lowest judgment”) was used to assess the feasibility of DESDE-LTC. This feasibility evaluation tool included three domains: Applicability, Acceptability, Practicality; plus another domain of Relevance (or criterion validity) [30]. This instrument was completed by 54 health service experts who participated in the nominal groups and researchers with previous experience in the use of ESMS/DESDE from Spain (n = 15), Slovenia (n = 10), Austria (n = 8), Bulgaria (n = 8), Norway (n = 6), United Kingdom (n = 3), Chile (n = 2), Germany (n = 1) and Italy (n = 1). This questionnaire is available online^b^. The consistency and usability of the Feasibility questionnaire was evaluated in the preliminary expert sample (21 respondents). This instrument obtained good internal consistency (Cronbach’s alpha over 0.7 in all domains) [32]. Only three questions out of 25 showed some problems of understanding. In the opinion of the experts, this questionnaire covered the main aspects of feasibility [33].

### Consistency

A content analysis of the hierarchy of the DESDE-LTC coding system was made by an ontology expert (MR-F) based on previous experience in the ontology analysis of other health classification systems [13]. Ontology analysis is not only relevant to the assessment of taxonomies and classifications but also for other standardized instruments [30]. The relationships of the different terms on the hierarchy were appraised according to their attributes. Two types of hierarchy were assessed depending on the attributes of the concept of interest: (1) structural assemble: <part-of> (part-whole), and similarity: <is-a> (kind-of). In a <part-of> hierarchy, terms inherit their location from parent terms higher in the hierarchical tree; (2) In a <is-a> (kind-of) hierarchy many different properties of parent terms are inherited by their children terms (e.g. a hospital <is-a> meso-organization <part-of> the global health system defined for a region or nation.). Additionally, a proxy quantitative analysis of the overall consistency or structural validity of the DESDE-LTC instrument was obtained by assessing the association of codes, stability and independence across three levels of the hierarchy using a Boolean Factor Analysis (BFA).

### Reliability analysis

170 minimal stable organizational units of health services (BSICs) covering the ‘Main Types of Care’ (MTCs) in Europe were selected by one member of the core group (MP) from the DESDE database and case vignettes provided by other European partners. All services were coded by two independent observers. The reliability analyses took into account both Classical Test Theory (CTT) and the Generalizability Theory (GT) [30]. Cohen’s kappa coefficient was used to provide a measure of the degree of agreement between two observers when both raters classified services into mutually exclusive categories. As part of the GT, a crossover design with two faces was used to obtain a measure of the dependability (reliability) between observers.

### Validity analysis

The validity analysis of the DESDE-LTC coding is a continuation of the previous studies on the validity of ESMS and DESDE. The development of this coding system has been a continuous iterative process following a bottom-up approach derived from the description and coding of hundreds of services for mental health, ageing and disabilities in different European countries and by different teams since the publication of ESMS [20]. This study describes the content validity of the taxonomy and the instrument to code MTCs (DESDE-LTC, Section B), its internal structure (construct validity), how its findings are judged as valid by different stakeholders including officers, managers and service researchers (criterion validity) and how this information could been used by policy makers (practical usability) [31].

Assessment of the face and content validity of the instrument is based on judgments made by the panel of 54 international experts using a series of questions incorporated to the feasibility questionnaire: (Section B, Applicability, Question B1: ‘In your opinion, is the data obtained when applying the instrument useful?’; Section E, Relevance; and Section B, Applicability, Question B3: ‘From your point of view, does the instrument cover important dimensions?’).

The quantitative validity of the DESDE-LTC instrument was analysed using a database comprising 1339 services (BSICs), assessed by the core group. The hierarchical structure of the instrument is composed of 4 levels: Level 0, composed of the 6 main branches (‘I’: Information, ‘A’: Accessibility, ‘S’: Self-support, ‘O’: Outpatient care, ‘D’: Day care, ‘R’: Residential care); Level 1, primary branches into which the main branches are divided; Level 2, intermediate level where some of the level 1 branches reach their final division while others sub-divide further; Level 3, final subdivision of branches. Each code in each level was assessed as present (1) or absent (0) in each of the services, resulting in a matrix of zeros and ones. Following thisprocess structure, the BFA approach was used to evaluate the internal structure at levels 0, 1 and 3 (level 2 was embedded in this final level). As in classical factorial analysis, BFA obtains, with dichotomous variables, a group of dichotomous factors to explain the underlying structure within the population. BFA adjusts the items observed to estimated ones by multiplying the factor loadings and the factor scores by the Boolean product [34]. Both positive and negative discrepancies are counted and the method gets the factors minimizing these discrepancies. Positive discrepancies are recorded when the observed rating is 1, while the analysis estimate is 0. Negative discrepancies are the number of times the observed rating is 0, while the estimate value is 1.

### Impact analysis

Finally, a preliminary impact analysis was carried out in three phases [5,35,36]: *Screening* (review of available instruments for description and coding of health services and literature on the topic with a focus on European Union); *Scoping* (Identification of scope at European, National, Regional and Local level at every participating country); and *Appraisal* (Evaluation of the taxonomy, instrument, webpage and training package using the mapping developed at the Scoping phase –Highest to lowest / 5-point likert).

## Results

A comprehensive taxonomy of health services based on ‘Main Types of Care’ (MTCs) has been developed through work on the previous instruments, ESMS and DESDE. Substantial changes have been incorporated into the new DESDE-LTC instrument, including the operational definition of BSIC, which is the minimal organizational unit that should be defined to allow comparisons of like-with-like in health services research, and the operational definition of the ‘Main Types of Care’, which are the codes that are assigned to every BSIC in DESDE-LTC. It is important to note that one BSIC can be described by more than one MTC. In our sample, 1339 BSICs corresponded to 1643 MTCs (252 BSICs have more than one MTC).

**Figure 1:**
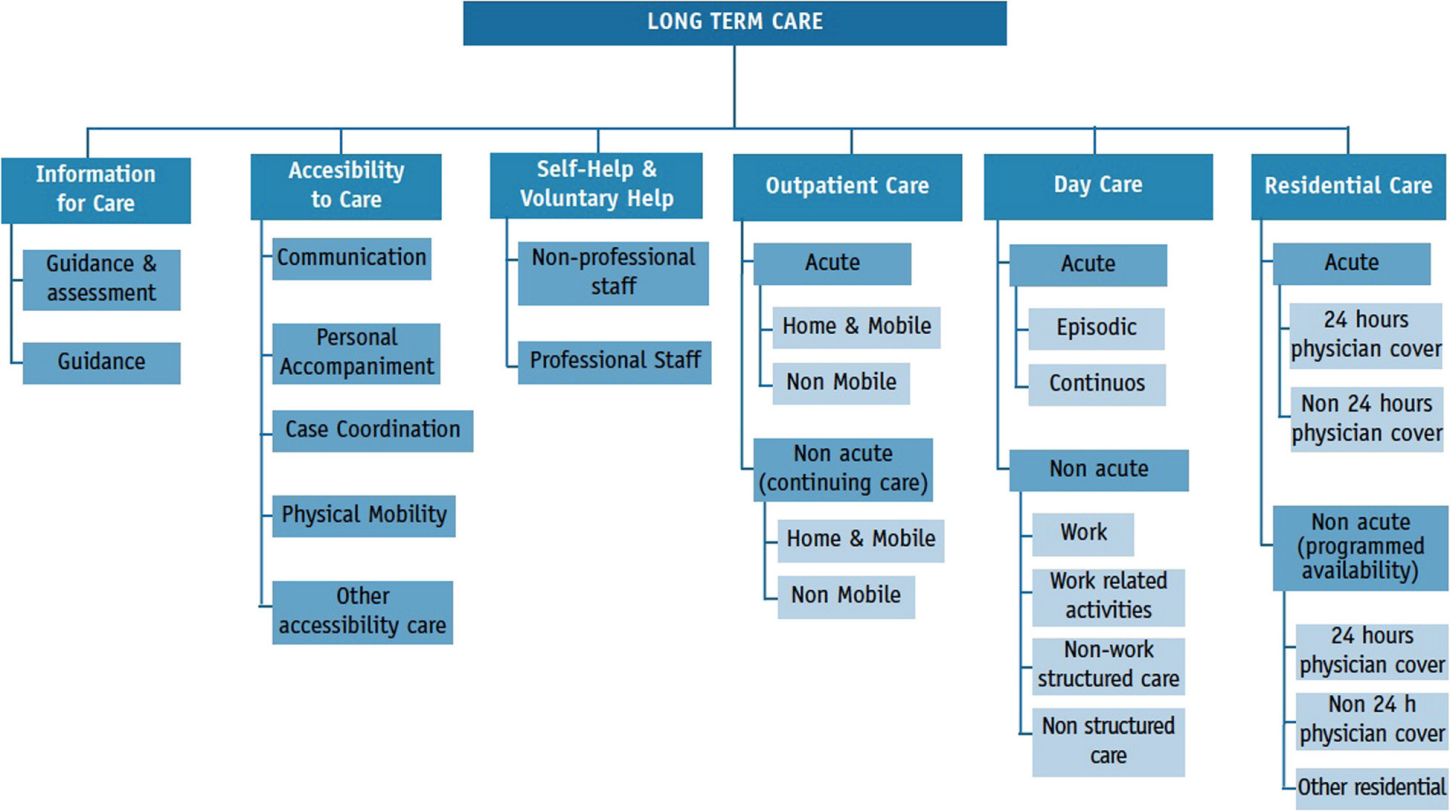
**Hierarchical structure of the version for Long Term Care (DESDE-LTC). **Tree structure of main and secondary branches.

The tree taxonomy has evolved from the original 4 main branches and 33 final codes used in the ESMS instrument to an ontology-driven comprehensive taxonomy of MTCs. According to the hierarchical tree structure of the instrument, level 0 is composed of the main 6 branches, level 1 is composed of the primary branches with 42 codes, level 2 is an intermediate level, and level 3 is composed of final subdivision of branches with a total of 89 codes that describe the characteristics of each object (Figure 1). Section A has incorporated eleven additional descriptors relevant for comparative policy analysis (for example the additional descriptor “h” is registered when outpatient care is provided at a hospital setting).

Each code is identified by three pieces of information, a Decimal Identifier, a Descriptor and a Label.

1. Decimal Identifier: this DI number encompasses every level of the hierarchy (first level the branch, second level acute/non acute, third level mobile/non mobile etc.). For example in the DI “D0203010100”, D stands for “Day care”, 02 stands for “non acute”, 03 indicates “non-work structured”, “01” high intensity, and 00 “no more levels of development”. This ID facilitates grouping the information for later statistical processing of data.

2. Descriptor: definition of the code according to the hierarchy (D4.1- Day care; non acute; non-work structured, high intensity).

3. Label (or MTC code): this is an internal code that groups all the characteristics described above for every final code (for example: D4.1). Not all the decimal identifiers have a label in the DESDE-LTC coding system. For example those decimal identifiers that correspond to intermediate branches of the taxonomy tree do not have a label assigned as they do not code actual services.

**Figure 2:**
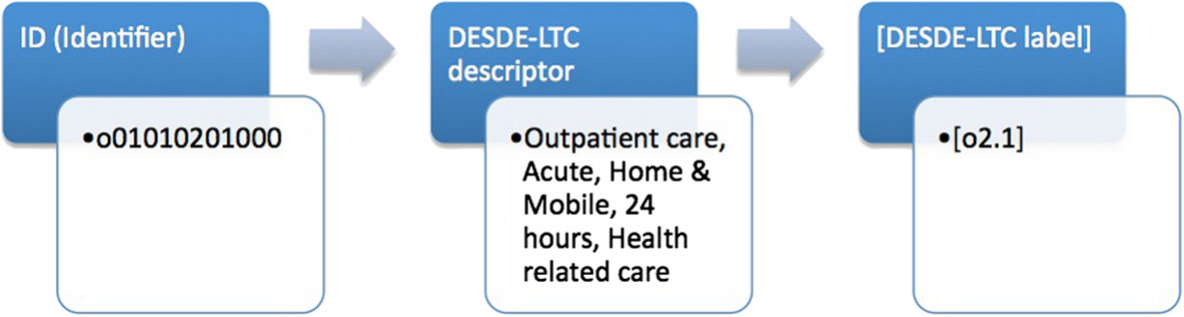
Main characteristics of the DESDE-LTC classification system: Decimal classification (Identifier), standard descriptor, and related label at the instrument.

The full alphanumerical coding and the glossary of terms are available on the webpage (http://www.edesdeproject.eu/download.php, see eDESDE-LTC Instrument). The labels are used at the instrument instead of the decimal identifier to facilitate its reading and its use (see example in Figure 2). The arrows in this figure indicate how the eDESDE-LTC coding reduces the complexity to understand and manage decimal identifiers and descriptors.

The DESDE-LTC instrument has four sections, like the previous questionnaire ESMS. Section A describes the general principles of evaluation and coding, the description of the catchment area and the target population of the services that should be coded in the mapping exercise. It includes the operational definition both of ‘Basic Stable Inputs of Care’ (BSICs) and ‘Main Types of Care’ (MTCs) and of the additional descriptors that are relevant for service comparison. Section B consists of a taxonomy tree that represents its hierarchical structure, the description of the codes, and their identification using MTC labels. Section C counts the levels of utilization of MTCs in a catchment area, and Section D describes the main characteristics of every service identified in the mapping exercise and coded in Section B (i.e. name, location, ownership, management, opening hours, staff, etc.).

### Feasibility analysis

DESDE-LTC fulfilled the criteria for feasibility on all four factors (best to good ratings). Applicability obtained an arithmetic mean of 2.1 (where 1 = “best/highest” and 5 = “worst/lowest judgment”). According to experts, data obtained using the instrument were very useful for understanding health care and LTC provision. The acceptability average rating was 2.3. As a result of the complexity of the systems in LTC, expert knowledge is considered an important precondition for use of this instrument. The information required for applying the instrument is difficult to obtain and often not readily accessible. Although the instrument was regarded as user-friendly, its underlying background, management and completion are not easy to understand due to many specific and new terms that require training and practical expertise, so more practical examples and exercises may be required. Ratings for practicality were less good (mean: 2.4).

The coding system and the analyses of data are quite complex and require advanced expert knowledge. In spite of these caveats, DESDE-LTC was judged as very useful in relation to the time and effort devoted to training and completion. Relevance (face validity) obtained the best mean rating (1.7). Finally, according to experts, the DESDE-LTC taxonomy and toolkit is a significant step forward towards achieving a classification of LTC services in the near future.

### Consistency: ontology and structure of the instrument

This classification contains a comprehensive and meaningful description of the terms of this domain which can be applied to eventually formalize an ontology based on the specification of the system (scope and purpose). The ontological analysis allowed for the development of a decimal systematic notation to facilitate a hierarchical scheme of services for long term care. The final taxonomy and toolkit reaches this objective in four different ways:

a) The taxonomy scheme of LTC services contains 89 decimal numeric identifiers (DESDE-LTC Classification). The decimal codification contributes to specifying the meaning of the represented objects, determining dependency relations of specific concepts to general concepts. This structure could be reused to formalize the accepted and shared knowledge encapsulated in DESDE-LTC in an ontology applicable to computer-based information systems.

b) The MTC label listing combines name and number of the tree taxonomy branches to provide a usable standard description of LTC services. Every label corresponds to a decimal identifier. This bi-univocal correspondence facilitates the precise meaning of the labels according to their position in the taxonomy.

c) The standard descriptor for every decimal identifier and its related label (DESDE-LTC Coding List) summarizes the main characteristics of each LTC service by its Main Type of Care, and facilitates a quick search of branches definitions.

d) The glossary of terms compiles an alphabetical list of definitions of key concepts that appear at DESDE-LTC classification and toolkit.

To assess the structural consistency of applicability of this taxonomy to code BSICs, a Boolean Factor Analysis (BFA) was run at three levels of the hierarchy (levels 0, 1 and 3) (see validity analysis below). The majority of the codes were explained by a single factor which indicates that codes are well defined and composed a consistent structure within the instrument. This analysis confirmed that main and secondary branches of DESDE-LTC are collapsed by codes (or items) that measure independent characteristics of the services being assessed.

### Reliability

A high level of inter-observer agreement was obtained when two independent observers assigned the codes to 170 services (BSICs). The reliability analysis for each code at levels 1 and 3 of the taxonomy tree (final branches) is shown at Table 2.

**Table 2:** **DESDE-LTC inter-rater reliability: Main Types of Care in main and final branches (*n*** = 435)^*^

**DESDE-LTC LABELS (MTC Codes)**	***n (Alfa + Beta)***	**Coef. G (GT) and K (CCT)**
Information for Care (I)	15	Coef. G: 0.95 (S.E.: 0.01917)
I1.1	2	κ*:* 1.00 (1.00-1.00)
I2	11	κ*:* 1.00 (1.00-1.00)
I2.1	6	κ*:* 0.79 (0.40-1.00)
I2.1.2	2	κ*:* 1.00 (1.00-1.00)
I2.2	1	*-*
Accessibility to Care (A)	17	Coef. G: 0.97 (S.E.:0.01088)
A1	2	κ*:* 0.66 (0.04-1.00)
A2	2	κ*:* 0.66 (0.04-1.00)
A4	12	κ*:* 1.00 (1.00-1.00)
A5	1	κ*:* 1.00 (1.00-1.00)
Self-help and Volunteer Care (S)	9	Coef. G: 0.80 (S.E.: 0.03321)
S1.2	5	κ*:* 0.79 (0.40-1.00)
S1.3	1	*-*
S2.1	2	κ*:* 1.00 (1.00-1.00)
S2.2	1	*-*
Outpatient Care (O)	120	Coef. G: 0.95 (S.E.: 0.0115)
O2.1	2	*-*
O3.1	18	κ*:* 0.64 (0.38-1.00)
O5.1	10	κ*:* 1.00 (1.00-1.00)
O5.1.1	6	*-*
O5.2.1	7	*-*
O5.2.3	2	κ*:* 1.00 (1.00-1.00)
O6.1	4	κ*:* 1.00 (1.00-1.00)
O6.2	2	κ*:* 1.00 (1.00-1.00)
O8.1	22	κ*:* 1.00 (1.00-1.00)
O9.1	35	κ*:* 0.96 (0.90-1.00)
O10.1	10	κ*:* 1.00 (1.00-1.00)
Day Care (D)	129	Coef. G: 0.97 (S.E.: 0.00769)
D1.2	27	κ*:* 0.95 (0.88-1.00)
D2.2	8	κ*:* 1.00 (1.00-1.00)
D3.2	16	κ*:* 0.93 (0.79-1.00)
D4.1	42	κ*:* 0.97 (0.92-1.00)
D4.2	4	κ*:* 1.00 (1.00-1.00)
D4.3	28	κ*:* 0.92 (0.81-1.00)
D8.3	4	κ*:* 0.79 (0.40-1.00)
Residential Care (R)	126	Coef. G: 0.99 (S.E.: 0.00362)
R2	20	κ*:* 1.00 (1.00-1.00)
R4	21	κ*:* 0.84 (0.67-1.00)
R5	15	κ*:* 0.93 (0.79-1.00)
R6	14	κ*:* 1.00 (1.00-1.00)
R8.2	4	κ*:* 1.00 (1.00-1.00)
R9	6	κ*:* 1.00 (1.00-1.00)
R11	27	κ*:* 0.95 (0.88-1.00)
R12	5	κ*:* 0.79 (0.40-1.00)
R13	14	κ*:* 1.00 (1.00-1.00)

^*^Coef. G Coefficient of Generalizability for main branches, *Coef. Κ* Kappa Coefficient for final branches.

The reliability between observers was high for the main branches (A, D, I, O, R or S) (κ = 0.9674 / CI: 0.9362; 0.9987). The two observers agreed on 14 codes in “A” (Accessibility), 51 in “D” (Day care), 2 in “I” (Information), 39 in “O” (Outpatient), 59 in “R” (Residential) and 1 in “S” (Self-help/volunteer). In the GT analysis the main branches were analyzed as different measurement conditions of a single facet. The reliability (or dependability) coefficient for one observer was G = 0.9322. For each main branch the primary subdivision or sub-branch (e.g. A1, A2, D0, D9, etc.) was taken into account and coded with ‘0’ (absent) or ‘1’ (present). The reliability was almost perfect (κ = 0.8-0.99) for all the branches.

The inter-rater reliability for the final branches (level 3) was calculated for 36 labels (MTC codes) which had a sufficient number of observations to calculate the Kappa coefficient. The agreement was strong (κ = 0.61-0.8) for ‘accessibility to care-communication and physical mobility’ (labels A1, A2), ‘outpatient acute non-mobile health related care’ (label O3.1), ‘self-help and volunteer care with non professional staff for accessibility to care’ (label S1.2), ‘low intensity social and culture structured care’ (label D8.3) and ‘residential with daily support’ (label R12). The agreement was nearly perfect for the rest of the codes, except for 5 codes in which there was no agreement because the observers assigned different codes. These codes were ‘non-interactive information’ (label I2.2), ‘self-help and volunteer care with non professional staff for outpatient care’ (label S1.3) and ‘self-help and volunteer care with professional staff for accessibility to care’ (label S2.2). There was no agreement for ‘outpatient home and mobile non-acute care, 3 to 6 days a week’ both related to health (label O5.1.1) and non-related to health (label O5.2.1).

In summary, the reliability was high for the main branches, sub-branches and final branches, except for a small number of labels (MTC codes) on Information, Self-support and special forms of Outpatient Mobile Care (see Table 2).

### Validity analysis

Table 3 shows the different levels of the hierarchy in this taxonomy, where 0 represents the main branches and 3 the final full description of the every MTC. It shows the codes that were found at least once in the sample of 1339 services (BSICs). Each code in each level was declared present (1) or absent (0) in each of the services, obtaining a matrix of ‘0’ and ‘1’ with 1339 rows by 6, 31 or 42 columns, according to the level 0, 1 or 3. 10 out of the 31 codes at level 1 are present less than 5 times, and 10 out of 42 codes at level 3 appear only once.

**Table 3:** Primary branches division (in parentheses, the codes found in the sample of 1339 services)


Level 0	Branch I	Branch A	Branch S	Branch O	Branch D	Branch R	Total
Level 1	2 (2)	5 (4)	2 (2)	10 (7)	9 (6)	14 (10)	42 (31)
Level 3	8 (5)	5 (4)	10 (4)	24 (10)	22 (9)	20 (10)	89 (42)

BFA was here used to determine whether the codes at levels 0, 1 and 3 actually evaluate independent characteristics of a service or whether they are redundant and can consequently be removed:

a) At level 0, it was not possible to explain the 6 branches with a number of factors smaller than 6.

b) At level 1, it is possible to obtain a 17-factor model that explains 95% of the codes present in the services (positive discrepancies: 4.3%). This model does not explain 11 codes (I1, A5, O6, O10, D2, R5, R6, R8, R9, R10 and R12), with the ‘R’ branch the worst adjusted of all. A more extensive 23-factor model still could not explain 6 codes with prevalence lower than 5 (positive discrepancies is 0.9%). Finally a 29-factor model explains all the the codes and shows 0% of positive discrepancy. This factor model showed an association between labels I1 and A5. The association between labels R2 (acute inpatient hospital care) and O3 (emergency care, 24 hours) remained constant for all the models. In factor 18, codes R2 and O3.1 appear together 114 times (100% of appearances). Facilities with hospital acute care (R2) commonly offer outpatient emergency care (O3.1).

c) A 24-factor model explains 95% of positive codes at level 3 (12 codes remain unexplained). Again the worst adjustment is shown at branch ‘R’. The 29-factor model explains all codes with a prevalence higher than 5 except for D4.2 (Table 4).

**Table 4:** MTC code associations in a 29-factor model (Boolean factor analysis)

**Summary:**				
*FACTOR 1*	I11	I12	I22	A5
*FACTOR 2*	D41			
*FACTOR 3*	I212			
*FACTOR 4*	A1			
*FACTOR 5*	A2			
*FACTOR 6*	A4			
*FACTOR 7*	I11	S12	D83	
*FACTOR 8*	O91			
*FACTOR 9*	O521	S13		
*FACTOR 10*	S21			
*FACTOR 11*	S22			
*FACTOR 12*	O81			
*FACTOR 13*	O511			
*FACTOR 14*	0523			
*FACTOR 15*	R13			
*FACTOR 16*	D12			
*FACTOR 17*	R11			
*FACTOR 18*	O31	R2		
*FACTOR 19*	R4			
*FACTOR 20*	O101			
*FACTOR 21*	I211			
*FACTOR 22*	D43			
*FACTOR 23*	D11			
*FACTOR 24*	D32			
*FACTOR 25*	D22			
*FACTOR 26*	R6			
*FACTOR 27*	O61			
*FACTOR 28*	R102			
*FACTOR 29*	R5			

Labels I1.1, I1.2, I2.2 and A5 are associated to factor 1. This association is mainly explained by the low prevalence of these codes in the database: 1 observation for I1.2, I2.2 and A5 and 3 for I1.1. This probably describes a very infrequent type of care in one service. Labels I1.1, S1.2 and D8.3 appear together in factor 7. Low intensity social and cultural structured day care (D8.3) is generally but not necessarily connected to volunteer care (non-professional staff-accessibility to care - S1.2). Factor 9 includes codes O5.2.1 and S1.3, which could also be explained by their low prevalence.

### Impact analysis

Decision makers and planners from key European organizations were contacted and invited to participate in a related international conference, as well as in DESDE-LTC meetings. A major practical output of this awareness strategy has been the incorporation of the DESDE in the list of references of the System of Health Accounts (SHA v2.0) edited by OECD, WHO and EUROSTAT [8]. The DESDE-LTC toolkit has recently been incorporated into a project aimed to study the financing, efficiency and quality of mental health system in Europe funded by the 7th framework (REFINEMENT project, 2011–2013).

Direct contacts with national/regional social and health planners have been made by all partners with active involvement of national agencies in Spain, Bulgaria and Slovenia and practical implementation in Spain. The DESDE-LTC toolkit and its coding system have been used to describe the Mental Health system in eight regions (or Autonomous Communities) of Spain, and it has generated knowledge for evidence-informed planning in four of them: Madrid, Basque Country, Cantabria [37], and Catalonia [38].

## Discussion

To our knowledge DESDE-LTC is the first taxonomy and related toolkit to assess services for long term care in Europe. This taxonomy incorporates the decimal coding system, the assessment instrument, a glossary of terms, a training package and other accompanying material in an open-access webpage (http://www.edesdeproject.eu). These documents include the preliminary version of the final results described here.

Terminologies and classifications usually precede and provide the framework for developing questionnaires and other health instruments [39]. DESDE-LTC has followed a reverse sequence: this classification was preceded by the development of instruments for assessing mental health services (ESMS) [20], services for elders (DESDAE) [40] and services for persons with disabilities (DESDE) [3]. Items initially designed to code MH services at section II of ESMS have evolved into a complex system of identification of basic and comparable arrangements of care organization (BSICs) with defined attributes and descriptors which are based on MTCs for patients with LTC needs. These categories have been populated in a series of studies funded by the European Commission, as well as by national and regional public health agencies, since 1994. The previous work has been revised and completed in the DESDE-LTC project by an international expert panel and 6 national nominal groups. Previous classifications were developed either for local and national coding, as a complement of health accounts (SHA 2.0) [8] or as a broad umbrella for coding the environmental context of care in the classification of functioning (ICF) [41].

The DESDE-LTC classification and its related toolkit have been evaluated as a promising tool, it is an ontology-driven system and is well adapted to the actual needs of health service research. Even though this taxonomy has been developed by a broad group of experts and stakeholders from different countries and diverse health and social care areas (ageing, chronic care, intellectual disability, physical disabilities, and mental health), several limitations should be mentioned.

First, the reliability and the quantitative validity analyses were carried out in a sample made mainly of mental health services in Spain. This fact was related to the direct involvement of the Spanish MH national and regional agencies in the use of the instrument for evidence-informed planning. Services from other sectors and other countries were added to the database, mental health is regarded as a paradigm of complex care including health, social, education, work and crime and justice services [42], and Spain has a high diversity of health and social care systems in its 17 regions [43]. Therefore, to be fully generalizable, our results should be completed by studies comparing different countries and carried out in different health sectors. Likewise the results of the reliability analysis should be completed with results obtained from other research groups and observers who did not participate in the development of the instrument. A pilot study comparing services in Sofia (Bulgaria) and Madrid (Spain) has recently being completed, and the usability of DESDE-LTC is currently being assessed in the comparison of 9 mental care systems in Europe (Refinement Project) [44].

Second, the on-line training package developed in this project could not replace advanced face-to-face training and it could only be used as complementary to traditional training where at least 24 training hours are required. Third, to facilitate DESDE-LTC implementation and to reduce training requirements it is necessary to develop a user-friendly computer version based on algorithms. Future developments include a version for mental health (ESMS-R: European Service Mapping Schedule Research Version).

The DESDE-LTC tool may have a significant impact in the assessment of efficiency and equity. It should be noted that the main domains of health equity are: i) Eligibility: Criteria for access to care services are equitable (specific groups are not excluded); ii) Availability: The care option is available in the catchment area; iii) Accessibility: The care option is not influenced by restrictions and/or limitations in time, distance or information (e.g. user rights); iv) Utilization: Available care alternatives are actually utilized by users; and v) Mobility: When moving to a new location users can access and utilize similar care alternatives to those available in the former location, or basic care alternatives are available and comparable across two different areas. To assess the different domains of equity DESDE-LTC provides a common terminology, a standard coding of LTC services, a standard procedure for data collection and meaningful comparisons across and within countries [13].

## Conclusions

The DESDE-LTC instrument is consistent with the principles of ontology. It thus represents a relevant contribution to establishing a common terminology, a taxonomy and coding of Long Term Care services in the European context, and a standard procedure for data collection and comparison. Even though this system is ready for use, its overall relevance will not be established until it has been used to develop integrated Health Information Systems and to assess service harmonization and allocation of care (resources, programs and interventions) between different groups and individuals in Europe.

## Endnotes

^a^These countries were: Austria (n = 5), Bulgaria (n = 5), Croatia (n = 1), Slovenia (n = 5) and United Kingdom (n = 4). To complement this initial study and the sample of assessed facilities a pilot study was carried out to compare the cities of Sofia and Madrid (http://www.edesdeproject.eu/reference.php).

^b^Available at the eDESDE-LTC website (http://www.edesdeproject.eu).

## Competing interests

The authors declare that they have no competing interests.

## Authors’ contributions

All authors have participated in different parts of the project eDESDE-LTC; LSC carried out the design and coordination of the eDESDE-LTC project; JAG participated in the revision of the project’ materials and in the writing of the final manuscript. All authors read, contributed and approved the final version of this text.

## Pre-publication history

The pre-publication history for this paper can be accessed here:

http://www.biomedcentral.com/1472-6963/13/218/prepub
